# From population to HIV: the organizational and structural determinants of HIV outcomes in sub-Saharan Africa

**DOI:** 10.1186/1758-2652-14-S2-S6

**Published:** 2011-09-27

**Authors:** Rachel Sullivan Robinson

**Affiliations:** 1 School of International Service, American University, 4400 Massachusetts Avenue NW, Washington, DC 20016-8071, USA

## Abstract

**Background:**

There exists no consistent explanation for why some countries are successful in combating HIV/AIDS and others are not, and we need such an explanation in order to design effective policies and programmes. Research evaluating HIV interventions from a biomedical or public health perspective does not always take full account of the historical and organizational characteristics of countries likely to influence HIV outcomes. The analysis in this paper addresses this shortcoming by testing the impact of organizational and structural factors, particularly those resulting from population interventions, on HIV outcomes at the country level in sub-Saharan Africa.

**Methods:**

The primary independent variables are factors that originated from efforts to slow population growth: whether a country has a long-time affiliate of the International Planned Parenthood Federation and whether a country has a population policy. Additional structural factors likely to impact HIV outcomes include the level of wealth, the level of cultural fractionalization, and the former colonial power. The present study uses multivariate regression techniques with countries in sub-Saharan Africa as the unit of analysis, and four measures of success in addressing HIV: the change in prevalence between 2001 and 2009; the change in incidence between 2001 and 2009; the level of overall antiretroviral coverage in 2009; and the level of antiretroviral coverage for prevention of vertical transmission in 2009.

**Results:**

Countries with the greatest declines in HIV prevalence and incidence had older International Planned Parenthood Federation affiliates and had adopted population policies, even after controlling for age of epidemic, level of antiretroviral coverage, and funding for HIV. Population policies are also important predictors of levels of overall antiretroviral coverage and of coverage of HIV-positive pregnant women to prevent vertical transmission. Structural factors with significant impacts include wealth, cultural fractionalization and former colonial power.

**Conclusions:**

The organizational and structural context of African countries is strongly predictive of HIV outcomes. This finding implies that policy and programmatic efforts should be put towards strengthening existing organizations and perhaps even creating new ones. The fact that cultural fractionalization also influences HIV outcomes suggests that efforts must be put towards identifying ways to reach political consensus in diverse societies.

## Background

There exists no consistent explanation for why some countries are successful in combating HIV/AIDS and others are not [1]. We need an explanation in order to design effective policies and programmes to address HIV/ AIDS, as well as to identify efficient allocations of scarce government and donor funds. The primary aim of this paper is to determine the factors that have allowed some countries to successfully combat HIV/AIDS, while other countries have struggled. Specifically, I test the hypothesis that strategies used in sub-Saharan African countries in the 1980s and 1990s to slow population growth impacted later success in reducing the prevalence and incidence of HIV, as well as in providing antiretroviral (ARV) therapy to HIV-positive individuals. In so doing, I emphasize the importance of macro contextual factors, including governmental policy and non-governmental organizations (NGOs), in determining HIV-related outcomes.

Until recently, there has not been much good news about HIV prevalence or incidence. And although prevalence and incidence rates are finally declining in many African countries [2], there still remains much work to be done in identifying the factors associated with successful responses to the epidemic, particularly in the realm of prevention. Using a case study approach, much of the existing research identifies political will and the capacity of African governments as predictive of HIV outcomes [3-8]. But some weak countries, like Uganda, have enacted positive change, while some of the richest countries, with the most capable governments, like Botswana and South Africa, have experienced persistently high prevalence rates.

The case study methodology thus fails to achieve the generalizations made possible by statistical analysis of more observations. Previously used methodological approaches therefore produce insufficient evidence for use in designing health-related interventions. To circumvent these issues, I rely on between-country variation in organizational and structural factors, particularly those associated with population interventions, in order to try to explain variation in HIV outcomes. Specifically, I analyze data on all sub-Saharan African countries to test for a statistically significant association between the organizational and political structures resulting from efforts to address population growth in the 1980s and 1990s, and HIV outcomes in the 2000s.

**Figure 1:**
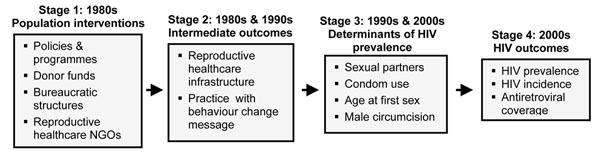
Conceptual model.

Figure 1 shows the model I test, which posits, in short, that countries that developed organizational and political structures related to providing family planning and moderating population growth were left with the resources and infrastructure necessary to mount more effective HIV/AIDS interventions. Stage 1 refers to governmental and social efforts to reduce population growth, which included national population policies and programmes designed to limit fertility, acquisition of donor funds for family planning, construction of physical and bureaucratic structures for providing contraceptive services and supplies, and the creation of local NGOs in the reproductive healthcare field. These efforts, which began as early as the 1960s in some countries, predated HIV/AIDS, which was not widely diagnosed until the mid-1980s.

The degree to which governments and societies engaged in such efforts influenced Stage 2 of the model, a set of intermediate outcomes that resulted from these efforts – domestication of techniques for behaviour change [9], governmental experience with donors, governmental and NGO familiarity with social mobilization efforts to induce behaviour change, and even family planning technologies (e.g., condoms) – that could then be translated into HIV-reduction efforts. These efforts, or lack thereof, in conjunction with factors ranging from culture to political economy to the status of women, then impacted the physical determinants of HIV prevalence, as shown in Stage 3, ultimately driving overall HIV outcomes in Stage 4.

The hypothesis that there exists a relationship between population-related interventions and later HIV outcomes rests on the assumption that many of the obstacles faced when implementing family planning programmes are similar to those experienced when implementing HIV prevention programmes. These include, and are not limited to, the challenges associated with talking about sex, particularly with young people, as well as concerns over altering sources of authority for sexual decision making. Simply put, preventing pregnancy and preventing HIV in sub-Saharan Africa both require that people change the way(s) they have sex. In both instances, governments, organizations and international actors with large sums of money have involved themselves, leading to a continuity of issues, actors and outcomes across interventions. For these reasons, we should see a relationship between earlier population interventions and later HIV outcomes.

This research adds to a small but growing body of literature addressing the links between population and HIV interventions. Stillwaggon [10] criticizes HIV interventions for paralleling population interventions and failing to address larger issues driving population growth and HIV transmission, specifically poverty. Richey [11,12] points to the continued narrow focus of population interventions, even in the era of reproductive health, on family planning, which comes at the expense of an integrated approach that includes HIV/AIDS. And Cleland and Watkins [9,13], while noting important differences between the two issues, state, “The ambitions, assumptions and implementation of both [population and AIDS] movements are strikingly similar and the social processes by which the AIDS crisis is ultimately resolved are likely to be similar to the processes that earlier led to the widespread adoption of fertility control” ([13], see page 208). This existing research, combined with the analysis in this article, supports the importance of analyzing the history of sex-related interventions in order to develop better policies and programmes, and ultimately improve human wellbeing.

The following section provides background on population interventions in Africa and the known determinants of successful HIV outcomes. I then discuss the examples of Senegal and Malawi, which illustrate the connections between the organizations and political structures associated with population interventions and later HIV outcomes, as a precursor to the statistical analysis that forms the core of the paper.

### Population interventions in Africa

Between the early 1960s, when most African countries gained independence, and the late 1970s, pregnancy prevention was not a primary concern of most African governments, organizations or individuals. A combination of economic and social motivations promoted high fertility norms at the individual level, and these were reflected at the national level by positive views towards population growth, which many African governments saw as a means to increase the size of their economies and to achieve scale efficiencies in production. During the 1960s and 1970s, private, non-governmental family planning organizations began to crop up in a number of countries, meeting the burgeoning demand for contraceptive services of primarily a well-to-do urban clientele, and by 1980, approximately half of African countries had such an organization [14]. By the 1980s, as recession loomed globally and donors promoted structural adjustment programmes and population reduction locally, some African governments began to view 2% to 3% annual population growth rates as a burden that challenged their promises to educate and employ citizens, as well as keep them healthy.

Population policies designed to limit population growth through reduced fertility were one result of this shift in perspective. Although Kenya and Ghana announced policies in 1967 and 1969, respectively [15,16], following these early declarations, there was an almost 20-year lag before a glut of policy announcements started in 1986 when Kenya announced a revised policy, and continued in 1988 when Nigeria, Senegal and Liberia adopted policies [17,18]. This trend continued through 1999, with 27 additional countries adopting new policies, and Ghana adopting a revised policy. Since then, no country out of the 15 remaining countries without policies has announced one, although some countries have revised their policies [19]. Generally speaking, these policies focus on reducing population growth as a means to achieve improved standards of living.

In addition to representing government willingness to address issues related to sex, population policies matter for a number of reasons. First, countries that adopted population policies received, on average, more funding from the United States Agency for International Development [20]. Second, countries with population policies experienced statistically greater fertility declines between 1987 and 2002 than those without such policies: 21% compared with 14% (author’s calculations from the World Bank [21]). Third, countries with population policies have a greater potential to improve gender and human rights because the policies motivate discussion of sex, generation and power, and provide language to groups promoting such rights [22].

### Determinants of successful HIV/AIDS outcomes

The key mechanisms through which reductions in HIV/ AIDS have been, and can be, realized are decreases in the number of overall and concurrent sexual partners, increases in condom use, increases in the age at first sex, and prevalence of male circumcision [23-32]. Existing scholarship has identified two main factors that operate through these mechanisms to determine country-level success in addressing HIV/AIDS: (1) political leadership and commitment; and (2) government coordination with NGOs and other civil society organizations.

Political commitment and leadership should help reduce HIV prevalence because they galvanize action around HIV/AIDS, organize those efforts, and provide legitimacy to messages promoting behaviour change [3-7,33-36]. There remains, however, no convincing cross-national study that shows that political commitment leads to reductions in prevalence of HIV, although factors such as lack of ethnic fragmentation [37] and press freedom, income equality and high HIV prevalence [38] lead to high levels of political commitment, and countries with “good” leadership provide better care to their HIV-positive citizens [39].

The second prominent factor associated with successful reductions in HIV prevalence is government interaction with civil society, broadly understood to include NGOs, community-based organizations, religious organizations, labour unions and other social groups [3,5-7,33-36]. Coordination with such groups provides the conduits through which messages about prevention are spread, as well as increases the perceived legitimacy of messages that cover sensitive issues relating to sex, morality and religion.

The most-frequently studied AIDS success stories in Africa are Uganda and Senegal. In Uganda, HIV prevalence declined from approximately 20% to 10% in the 1990s [30]. The mechanisms for Uganda’s decline were a decrease in number of sexual partners and an increase in condom usage [30,40]. The drivers for these changes included political leadership on the part of the country’s charismatic president, Yoweri Museveni, a decentralized government that allowed for local experimentation and personalization of responses to HIV/AIDS, and active incorporation of different social groups in prevention efforts [6,23,40-43].

In Senegal, HIV prevalence has remained at approximately 1% since the 1980s [23]. The mechanisms for this lack of increase in prevalence include low numbers of multiple concurrent sexual partners, a less virulent form of the virus (HIV-2), an increase in the age at marriage and first sex, and almost universal male circumcision [7,8]. These outcomes resulted from early government acknowledgement of HIV, effective management of sexually transmitted infections among sex workers, and active incorporation of social groups, particularly religiously oriented ones, in distributing HIV-prevention messages [8,23,34,42,44-47].

The emphasis of the literature on political commitment, and on the cases of Senegal and Uganda, poses three challenges to determining the causes of variation in country-level success addressing HIV/AIDS. First, political commitment is a difficult variable to measure [7,34,35,48] and may not actually translate into action once countries have learned that displays of political commitment are necessary to garner and maintain international support [42,46]. Second, the cases of Senegal and Uganda do not generalize well. In Uganda, the timing of the decline in HIV prevalence indicates that behaviour change most likely occurred *prior* to intervention by Museveni and international donors, and so is probably not the result of policy [1,30,49]. In Senegal, there is no way to know whether the epidemic would have actually grown out of control in the absence of the government actions taken [50], particularly given the relative protection provided to the population by near-universal male circumcision and other factors.

The third challenge to determining the causes of variation in country-level success addressing HIV/AIDS is that although the literature has identified government engagement with civil society as key to fighting HIV/AIDS, no systematic research has incorporated measurements of the strength of civil society. The analysis that follows addresses all three challenges to the existing literature by testing a new hypothesis about the legacy of population interventions, employing a multi-country analysis, and incorporating information on the historical depth of NGOs. These contextual factors are highly likely to impact HIV-related outcomes.

### The examples of Senegal and Malawi

Population and HIV interventions in both Senegal and Malawi provide support for the hypothesis that experience with country-level population interventions impacted later success in addressing HIV/AIDS. In Senegal, there is evidence that some of the factors associated with its successful response to HIV mirror previous experience gained in response to population issues, including the development of NGOs, government support of sex-related health issues, and government and NGO interaction with religious leaders. In Malawi, there is evidence that *negative* experiences with population interventions may have spilled over onto early HIV efforts. I provide these cases to illustrate the potential causal pathways through which the variables representing population interventions included in the statistical analysis that follows (early family planning NGOs and the existence of a population policy) may have influenced HIV outcomes.

As mentioned, Senegal was a vanguard population policy adopter in 1988. While the policy resulted from an intersection of national and donor goals, practically it represented the willingness of the government to address issues related to sex. In addition, through the 1980s and mid-1990s, Senegal ranked in the top third of African countries based on the degree of effort put towards providing family planning services and supplies [51]. These government efforts were rewarded by donors, as Senegal was a popular recipient of international aid for population activities [52].

In addition to positive government efforts towards family planning, Senegal’s strong civil society encompassed a number of reproductive health NGOs. In 1985, prior to the emergence of HIV/AIDS, there were 31 local NGOs doing some work in the area of reproductive health in Senegal [19], and this figure grew to 57 by 1989. One of the most important NGOs involved in family planning and sexual health in Senegal, the *Association Sénégalaise pour le Bien-être Familial*, or ASBEF, was founded in 1975 and affiliated with the International Planned Parenthood Federation in 1981. It provides sexual and reproductive health services, particularly contraception, to youths as well as to women through clinics in the majority of Senegal’s regions.

NGOs helped facilitate dialogue on population issues with religious leaders, civil society and the government. A national seminar entitled “Islam et Population” was held in 1984, with the assistance of ASBEF [53]. ASBEF also hosted a roundtable on Islam and family planning in 1989. Then, following the 1994 United Nations conference on population and development held in Cairo, Egypt, a set of networks related to religion and contraception were formed. One was the *Réseau des Parlementaires Sénégalais pour la Population et le Développement* (Senegalese Network of Parliamentarians on Population and Development), making Senegal the first country to have a network of parliamentarians working on population issues.

The existence of ties between the government and religious leaders, and between NGOs and religious leaders, also proved to be beneficial in response to HIV. Specifically, outreach to civil society organizations, particularly religious ones, began with efforts to promote family planning, and most likely spilled over into HIV prevention efforts. Senegalese government coordination with religious leaders on HIV dates from at least 1989, and in 1994, the primary US-funded AIDS programme in Senegal, AIDSCAP, and the Senegalese government surveyed religious and political leaders regarding their attitudes towards AIDS [54]. One of the recommendations from the analysis of this data was a national colloquium on religion and HIV, as religious leaders had indicated that they wanted to be involved in the response to AIDS [54], and this role was institutionalized with a major conference in 1995 between Muslim and Christian leaders [55].

An additional set of parallel conferences were held on religion and HIV. The first, in 1995, was entitled “AIDS and Religion: The Response of Islam” [54], and was attended by Islamic religious leaders from all over the country. In addition to providing an important opportunity for dialogue, the key outcome from the conference was a statement that it was acceptable for serodiscordant couples to use condoms [54]. The second, “AIDS and Religion: Responses of Christian Churches” was held in 1996, and was also attended by Islamic leaders [54]. Although there is no explicit evidence that religious leaders’ practice of dialoguing about family planning paved the way to similar conversations about HIV, the parallel experiences are certainly suggestive.

Malawi’s response to population growth differed dramatically from that of Senegal. Hastings Kamuzu Banda, president from 1964 to 1994, exercised a form of authoritarian rule that emphasized cultural nationalism, particularly respect for hierarchy and authority [56]. As a result, he found western “permissiveness” particularly threatening, and had a very narrow view of the role of women [56], both of which made family planning unacceptable and led him to ban it during the 1960s [15]. As donor interest in family planning increased in the 1980s, the Malawian government remained unwilling to fully endorse family planning, and so implemented a “child-spacing” policy in 1982 with a goal to increase the number of years between births [15]. It was not until Malawi transitioned to democracy and Banda left office in 1994 that the government adopted a national population policy [15].

In parallel, through the 1980s, Malawi had family planning effort scores in the bottom third of African countries, and had only moved to the middle tier by the mid-1990s [57]. The primary family planning organization in the country, *Banja La Mtsogolo*, was not founded until 1987, and the affiliate of the International Planned Parenthood Federation (IPPF) only came into existence in 1999 [19] when the government parastatal involved in family planning, the National Family Welfare Council, was privatized ([58], see page 18).

Malawi’s initial response to HIV/AIDS was mixed. Despite being a medical doctor, President Banda had minimal commitment towards HIV/AIDS [43]. The Ministry of Health’s National AIDS Control Programme, started in 1987 [34], was ultimately quite ineffective [43]. AIDS was declared a national emergency in 1999, but this still did not provoke much local interest, and the removal of the National AIDS Control Programme from the Ministry of Health in 2001 in order to comply with World Bank guidelines, decimated the ministry and further hampered efforts to address HIV [34]. Surface efforts to address HIV/AIDS continued: a national AIDS policy followed in 2004, and that same year, HIV became a campaign issue for the first time [43]. It was not really until ARV therapy became widely available in 2004 [59], however, that the intensity of the response to HIV skyrocketed in Malawi.

The fact that the government began to care about population growth at the same time as HIV/AIDS was leading to increased mortality made the government’s efforts in relationship to HIV all the more suspect [60]. Like family planning, HIV was also viewed as something dubious that came from abroad [61]. Family planning was seen as a western effort to take the fun out of sex, as were the condoms that health workers and NGOs insisted be used to protect against HIV. As a result, the acronym for AIDS was given an alternative interpretation: the American Invention Depriving Sex” [61].

Amy Kaler [60] has explained the suspicion about AIDS and condoms as the “long shadow of population control” describing how everyday Malawians’ interpretation of family planning efforts impacted their understanding of AIDS and AIDS interventions. Specifically, Malawians interpreted family planning efforts as the combined efforts of donors and the government to decimate the population of a country that was constantly begging for international aid. Given this degree of suspicion about population control, when the same actors began talking about AIDS, Malawians saw AIDS as a continuation of those population control efforts: a further concerted effort to eliminate the population. Because the same actors also proposed solutions for AIDS, particularly condoms, Malawians were understandably suspicious. As a result, condoms were viewed as dangerous, ineffective and possibly even the source of AIDS itself.

The examples of Senegal and Malawi suggest that the nexus of interventions related to population growth and family planning both practically and symbolically structured the macro context in which these same countries addressed HIV. I turn to testing this hypothesis in the following statistical analysis, which looks particularly at whether countries had an early affiliate of the IPPF (which Senegal did and Malawi did not), as well as whether countries had a population policy.

## Methods

The objective of the analysis is to determine how government and social efforts to slow population growth in the 1980s and 1990s in sub-Saharan African countries impacted HIV outcomes in the 2000s. These population-related efforts included population policies and reproductive healthcare NGOs. I next describe a unique data set that I constructed covering all sub-Saharan African countries that captures the institutional context, much of it built from population interventions, relevant to HIV outcomes.

I measure HIV outcomes in four ways: the change in adult HIV prevalence between 2001 and 2009; the change in adult HIV incidence between 2001 and 2009; the level of overall antiretroviral coverage in 2009; and the level of antiretroviral coverage for HIV-positive pregnant women to prevent vertical transmission in 2009. All data come from UNAIDS [2]. While the ideal dependent variable for assessing the impact of prevention interventions is change in incidence rates [1,24,62], I include an analysis of both prevalence and incidence rates given that greater uncertainty exists around estimates of incidence than around estimates of prevalence. As the analysis covers only sub-Saharan Africa, where the vast majority of countries have generalized epidemics, this means that national figures are estimated primarily from data from pregnant women attending antenatal clinics [63]. As estimates, these figures are subject to error resulting both from input data and the assumptions of the models themselves [64].

Despite these drawbacks, according to the most recent assessment [65], input data for sub-Saharan Africa are of generally high quality. Of the 45 countries for which data quality assessments exist for both 2001 and 2009, only seven were rated as having “poor” data quality both years [65,66], and approximately half of those countries were ultimately removed from the analysis because of missing data. Furthermore, UNAIDS’ models have been improved and refined over time [2,63], such that the data used for the analyses in this paper, although not perfect, are of the best quality available.

Data on the reported number of people receiving and needing antiretroviral therapy in 2009 are based on the World Health Organization’s 2010 guidelines, which include a lower cutoff to identify need for therapy than had been used up until that point [2]. The percentage of HIV-positive pregnant women receiving antiretroviral therapy at the time of birth also comes from the same report, and I refer to this as prevention of mother to child transmission (PMTCT) coverage [2].

Variables that capture the degree of policy and organizational resources available to a country in addressing HIV/AIDS include the following:

1. Existence of population policy. This comes from the various population documents themselves, the *Annual Review of Population Law *[*18*] and the United Nations Population Fund and Population Reference Bureau [17,67] publication, *Country Profiles for Population and Reproductive Health: Policy Developments and Indicators.*

2. Early IPPF affiliate. This indicates whether the IPPF had an affiliate in a given country before 1986 [14] as this date marks the point at which many African countries had their first diagnosed case of AIDS. All but four countries (Equatorial Guinea, Sao Tomé et Principe, Somalia, and Zimbabwe) had an affiliate by 2009. While some of these organizations were directly set up by IPPF, others were in existence already and then affiliated with the IPPF later, like ASBEF in Senegal. The earliest organization was founded in 1932 (South Africa) and the latest in 1999 (Malawi).

Variables that capture factors not directly related to organizations and policy, which might still influence HIV outcomes, include:

1. Economic wellbeing. This is proxied by gross domestic product (GDP) per capita, from the World Development Indicators [68], and averaged over the period 2001-2009.

2. Cultural fractionalization. This is taken from Lieberman [37] and is originally from Fearon [69]. Cultural fractionalization is measured as the probability that two people drawn randomly from the population will be from two different groups as defined by ethnicity and language, particularly emphasizing linguistic difference. It ranges from 0 (no diversity) to 1 (complete diversity) and is a Herfindahl Index calculated as  where *s_i_.* is the share of group *i* out of N total groups. Easterly and Levine [70] and Lieberman [37] have shown that more diverse countries have worse outcomes because of challenges that such diversity poses to the allocation of public goods.

3. Former colonial power. This is from Bratton and Van de Walle [71], and is operationalized as a binary indicator for whether the country was a former British colony. This variable serves as a proxy for institutions, as well as language.

Other variables likely to directly influence the degree of decline in HIV prevalence and incidence include:

1. PEPFAR focus country. This indicates whether a country was one of the 12 sub-Saharan African countries that were part of the original 15 focus countries of the US President’s Emergency Plan for AIDS Relief (PEPFAR). This variable thus indicates the receipt of large amounts of HIV-related funding starting around 2003.

2. Funding received from the Global Fund to Fight AIDS, Tuberculosis and Malaria. This is the total funds specifically for HIV activities disbursed to a country from the Global Fund between 2002 and 2009, divided by the country’s average population size during the period to create a per capita measure [72]. All countries except Cape Verde received some funding for HIV from the Global Fund during this time period.

3. Antiretroviral coverage (2006). This is a measure of the proportion of those HIV-positive individuals in 2006 in need of antiretroviral coverage who were receiving it, from the World Health Organization’s online database [73]; 2006 is the intervening year for which the most complete data are available and refers to the old cutoff for assessing need for ARVs. Better provision of antiretroviral therapy is likely to make HIV prevalence higher because it reduces mortality, and thus increases the number of HIV-positive individuals in a population.

4. Epidemic peak. This variable is a measure of whether a country’s HIV epidemic peaked before 1999 and is based on the epidemic curves presented by UNAIDS and WHO in their epidemiological fact sheets for each country in sub-Saharan Africa [74]. Countries whose epidemics have plateaued are coded depending on whether the plateau was reached before or after 1999. Countries with older epidemics have a greater chance of experiencing a decline in prevalence because of more time for the epidemic to run its course, or for the government and society to respond.

I experimented with a number of variables that were ultimately not utilized in the name of parsimony, and because their absence does not substantively change the results. These included whether a country had experienced a war and whether a country was a democracy, as well as a finer specification of former colonial power, including an indicator for former French colonies. Similarly, although controlling for the number of births attended by a skilled practitioner is a reasonable control for the analysis of PMTCT coverage, skilled birth attendance is highly correlated with GDP per capita (Pearson’s r=0.704), so the inclusion of GDP per capita in the model is sufficient. An additional control that would be ideal to include, but for which data are too extensively missing, is death rates from HIV, which can reduce prevalence in the same way that antiretroviral coverage can increase prevalence. Finally, although family planning effort scores [57] capture some of the dynamic I describe in terms of population interventions, they are missing for far more countries than either the IPPF affiliate or population policy data, which exist for all countries.

**Figure 2:**
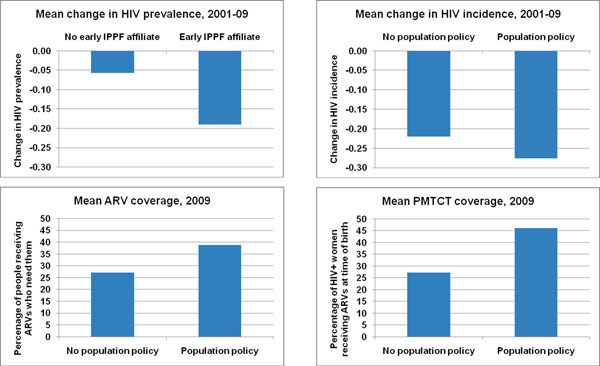
**Comparison of organizational and political variables related to population interventions with HIV outcomes.** IPPF, International Planned Parenthood Federation; ARV, antiretroviral; PMTCT, prevention of mother to child transmission.

I discuss univariate and bivariate statistics first, and then present the results from multivariate, ordinary-least squares regressions predicting the change in HIV prevalence between 2001 and 2009, the change in HIV incidence between 2001 and 2009, ARV coverage in 2009, and PMTCT coverage in 2009. The data set theoretically has 47 observations, representing each country in sub-Saharan Africa, but due to missing data and several outliers, analyses have between 32 and 42 countries. A larger sample size would be ideal, but the lack of reliable, comparable data on HIV prevalence or incidence from before 2001 or between 2001 and 2009, makes a country-year analysis impossible. Similarly, while expanding the analysis to look at all developing countries would be feasible, given the unique dynamics and timing of population interventions in sub-Saharan Africa, it makes the most sense to test the hypothesis on Africa alone. The small N indicates in particular that the multivariate results should be interpreted in conjunction with the case studies described here, as well as the bivariate results in Figure 2.

## Results

Table 1 shows descriptive statistics for all variables included in the analysis. On average, HIV prevalence declined by 13% between 2001 and 2009, and HIV incidence declined by 26%. These averages, of course, obscure a good deal of variation, with change in HIV prevalence ranging from a decline of 48% (Côte d’lvoire) to an increase of 25% (Guinea-Bissau), and change in HIV incidence varying from an 81% decline (Namibia) to a 4% increase (Uganda). In terms of the other two dependent variables, on average 36% of people needing ARV therapy in 2009 received it, while 42% of HIV-positive pregnant women on average received PMTCT interventions.

**Table 1:** Descriptive statistics

Variable	Mean	Std. dev.	Min.	Max.	N
Dependent variables					
Change in HIV prevalence 2001-2009	-0.13	0.19	-0.48	0.25	34
Change in HIV incidence 2001-2009	-0.26	0.23	-0.81	0.04	32
Antiretroviral coverage, 2009	35.7	19.5	2.0	88.0	42
PMTCT coverage, 2009	41.6	26.8	2.0	95.0	41
Population-related variables					
Population policy indicator	0.78	0.42	0	1	34
IPPF affiliate founded before 1986	0.63	0.49	0	1	34
General controls					
Average GDP per capita, 2001 -2009 (2000 US$)	821	1,081	141	4,059	34
Cultural fractionalization	0.42	0.19	0.00	0.73	34
Former British colony	0.41	0.50	0	1	34
HIV-related controls					
PEPFAR focus country	0.34	0.48	0	1	34
Average per capita Global Fund HIV disbursements, 2001 -2009 (US$)	9.76	13.20	0.77	63.98	34
Antiretroviral coverage, 2006	0.29	0.21	0.06	0.95	34
Epidemic peaked prior to 1999	0.31	0.47	0	1	34

Sources: See text

Note: Descriptive statistics for dependent variables refer to the countries included in the analysis of that variable. Values for all other variables refer to the analysis of change in prevalence (N=34). The descriptive statistics are highly similar, however, regardless of the particular sample. See note at bottom of Table 2 for detailed listing of countries excluded from each sample.

In terms of independent variables, almost 80% of countries have a population policy, and slightly less than two-thirds of countries have an IPPF affiliate founded before 1986. On average, countries had a GDP per capita of slightly more than $800 per year. Cultural diversity is relatively high, with an average fractionalization score of 0.42, and 40% of countries are former British colonies. In terms of the HIV-related controls, a third of countries are PEPFAR focus countries, and countries received on average slightly less than $10 per person from the Global Fund between 2002 and 2009. Finally, in 2006, only slightly more than a quarter of people in need of antiretroviral therapy were receiving it (based on the old, WHO guidelines), and the epidemic peaked before 1999 in approximately one-third of countries.

Figure 2 depicts the bivariate relationships between the key population interventions (early IPPF affiliates and the existence of a population policy) and the dependent variables. All of these figures show effects in the expected direction: having an early IPPF affiliate or a population policy is associated with better HIV outcomes. The difference is statistically significant at the p <0.05 level for the change in HIV prevalence, and at the p <0.10 level for ARV and PMTCT coverage.

Table 2 presents the results from the multivariate analysis, which includes four different dependent variables and similar independent variables. Models 1 and 2 present standardized coefficients from ordinary least squares (OLS) regressions predicting change in HIV prevalence and change in HIV incidence, respectively, between 2001 and 2009. A decrease in the dependent variable is a *good* thing, so negative coefficients indicate a more favorable outcome (a larger decline in prevalence or incidence). Models 3 and 4 present standardized coefficients from OLS regressions predicting levels of ARV and PMTCT coverage in 2009. In these models, positive coefficients indicate a more favourable outcome (greater coverage).

**Table 2:** Standardized coefficients from ordinary least squares regressions predicting HIV outcomes, sub-Saharan Africa, 2001-2009

Covariates	(1) Change in HIV prevalence 2001-09	(2) Change in HIV incidence 2001-09	(3) ARV coverage 2009	(4) PMTCT coverage 2009
Population-related variables				
Population policy indicator	-0.102	-0.533*	0.303*	0.304**
IPPF affiliate founded before 1986	-0.468*	-0.025	-0.009	-0.050
General controls				
GDP per capita	-0.027	-0.671**	0.271*	0.347**
Cultural fractionalization	-0.196	0.120	-0.261 *	-0.124
Former British colony	0.330	0.454*	-0.214	0.202†
HIV-related controls				
PEPFAR focus country	0.365†	-0.187	0.349*	0.301**
Global Fund HIV disbursements	0.089	-0.527*	0.373**	0.359**
Antiretroviral coverage	-0.361	0.455†		
Epidemic peaked prior to 1999	-0.549**	-0.391 *		
N	34	32	42	41
R^2^	47.9%	46.4%	56.1 %	72.9%

Note: Significance indicated by † p < 0.10 level; * p < 0.05 level; ** p < 0.01 level, *** p < 0.001 level

Countries missing from Model 1: Cape Verde, Comoros, Democratic Republic of the Congo, Equatorial Guinea, Ethiopia, Gambia, Liberia, Mauritius, Sao Tome and Principe, Senegal, Sierra Leone, Somalia and Sudan

Countries missing from Model 2: Burundi, Cape Verde, Chad, Comoros, Democratic Republic of the Congo, Equatorial Guinea, Ethiopia, Gambia, Liberia, Madagascar, Mauritania, Mauritius, Sao Tome and Principe, Somalia and Sudan

Countries missing from Model 3: Cape Verde, Comoros, Equatorial Guinea, Sao Tome and Principe, and Somalia Countries missing from Model 4: Cape Verde, Comoros, Equatorial Guinea, Gabon, Sao Tome and Principe, and Somalia

Model 1 shows that after controlling for whether a country has an “old” epidemic (one that peaked prior to 1999), as well as antiretroviral coverage, the best predictors of declines in HIV prevalence are having an early IPPF affiliate and being a PEPFAR focus country. Holding all variables constant at their means, a country with an early IPPF affiliate is predicted to experience a 20.6% decline in HIV prevalence between 2001 and 2009, while a country without an affiliate is expected to have only a 3.0% decline. The positive sign on the PEPFAR variable indicates that focus countries experienced smaller declines in prevalence than non-focus countries. This finding is most likely not reflective of the impact of PEPFAR’s activities, but rather the result of PEPFAR generally targeting hard-hit countries.

Model 2 shows that many more factors are predictive of change in HIV incidence than of change in HIV prevalence. Specifically, having a population policy, as well as more GDP per capita is associated with greater declines in incidence. Specifically, holding all variables constant at their means, a country with a population policy would be predicted to experience a 32.7% decline in HIV incidence between 2001 and 2009, while a country without such a policy would be predicted to experience only a 3.8% decline. Furthermore, in this model, Global Fund disbursements play a positive role (leading to greater declines). Intriguingly, the coefficient for former British colonies is significant but positive, indicating that former British colonies experienced *smaller* declines in incidence. While it is possible that this outcome reflects some difference in these countries’ institutional capacity resulting from colonialism, more likely it is the result of the fact that most former British colonies are located in southern and eastern Africa, the areas hardest hit by the HIV epidemic.

Models 3 and 4, predicting different types of antiretroviral coverage, are largely consistent with one another. Countries with population policies do a better job of providing such services to their citizens. Indeed, a country with a population policy is predicted to have an ARV coverage rate 13.3 absolute percentage points higher, and a PMTCT coverage rate 18.7 absolute percentage points higher, than a country without such a policy, holding all other variables constant at their means. In addition, wealthier countries also more effectively provide antiretroviral coverage. Interestingly, in the case of overall ARV coverage specifically, high levels of cultural fractionalization are associated with lower levels of coverage, which echoes the findings of Lieberman [37]. The effect of international funding for HIV-related activities clearly comes to bear in these models, as PEPFAR and Global Fund funds are both positive predictors of antiretroviral coverage.

The four models are designed to capture two types of success in addressing HIV. Models 1 and 2 analyze factors that may be associated with successful prevention efforts, while Models 3 and 4 are solely about treatment. Looking at the models in this way suggests two key observations. First, it seems to be more difficult to predict changes in HIV prevalence and incidence than it is to predict treatment success: the R^2^ values are somewhat lower for the first two models than for the second two models. This means that there are additional factors, most likely difficult-to-measure ones, driving differential success in prevention efforts.

Second, resources (broadly construed) are clearly very important to both prevention and treatment. Greater amounts of GDP per capita are associated with better prevention *and* treatment options. This may indicate that there are actually more resources to be put towards interventions, that there exist other social institutions that similarly facilitate interventions, or may reflect lower levels of poverty, which can drive HIV outcomes through numerous pathways. And while there is some evidence that greater resources in the form of more foreign aid directly targeting HIV is associated with greater prevention success, funds from PEPFAR and the Global Fund are strongly associated with treatment success, indicating the challenges of prevention interventions.

Omitted variables are an important consideration in any statistical analysis. Two hypothetically important variables, democracy and conflict, were knowingly omitted from the regression analysis in the name of parsimony because they showed no correlation with any of the dependent variables in either bivariate or multivariate contexts. There are, however, other variables that may influence the transmission of HIV and so may also particularly influence Models 1 and 2, but that were not entered into the regressions. These include variables related to the prevalence of parasitic infections, including malaria, schistosomiasis and various intestinal helminths [10,75], as well as variables measuring exposure to HIV through unsafe injection practices [75].

While rigorous debate continues about the relative role of these factors in explaining variation in HIV prevalence [76,77], their potential impact is partially reflected by the inclusion of GDP per capita. Inclusion of alternative measures for GDP per capita that relate to different theories about the transmission of HIV (the percentage of the population living on less than two dollars per day, the percentage of the population with access to clean water, and the percentage of the population under-nourished) yielded substantively similar results for the analysis of change in HIV prevalence, and were much less predictive than GDP per capita in the analysis of change in HIV incidence (results not shown).

Taken together, these models show a significant impact of organizational and political factors resulting from population interventions – specifically, IPPF affiliates and population policies – on HIV outcomes. They also indicate that other factors are important. Level of resources, both in the form of GDP per capita and in the form of funds from major HIV donors, are significantly associated with positive HIV outcomes, particularly those related to provision of antiretroviral coverage. Cultural fractionalization plays a role in overall ARV coverage, while the role of being a former British colony seems to be mixed. In the case of change in HIV incidence, it is associated with worse outcomes (perhaps because this variable also captures southern African countries), while in the case of PMTCT coverage, it is associated with better outcomes (perhaps because of better institutional structures).

## Conclusions

The analysis in this article provides support for the hypothesis that interventions related to slowing population growth that predated the HIV epidemic in sub-Saharan Africa have impacted HIV outcomes. Given that this is a statistical analysis, the causes of this relationship cannot be definitively ascertained. The brief discussion of Senegal and Malawi, however, provides more details on the causal mechanisms at play. Specifically, it seems that in Senegal, the development of NGOs, as well as government interaction with religious leaders associated with efforts to provide family planning, may have laid the groundwork for similar relationships addressing HIV/ AIDS. In Malawi, however, suspicions about condoms and outside efforts to change sexual behaviours that dated from the time before HIV/AIDS may have made an already difficult task, HIV prevention, even more difficult.

The quantitative analysis of all sub-Saharan African countries reinforces this interpretation. Having an older IPPF affiliate is associated with better HIV outcomes. Most likely, IPPF organizations were able to move fairly seamlessly into HIV-related work from their family planning base. They may also have had experience accessing both hard-to-reach populations, as well as targeting powerful social actors, such as religious leaders and politicians. The interpretation of the population policy variable is somewhat less straightforward. Having one can be interpreted as representing governmental willingness to address issues related to sex, good relationships with donors, or even government effectiveness (i.e., the ability to pass policies).

In addition to supporting the hypothesis that historical factors related to previous health interventions have impacted HIV outcomes, the findings from this analysis also touch on a current debate, which is about the relative merits of further integrating family planning and broader reproductive healthcare services into HIV interventions. There is solid rationale for attempting such integration [78], given that condoms prevent both pregnancy and HIV transmission, and because family planning is frequently the only way that African women interact with the healthcare system [79]. While much of this discussion relates to service provision, this analysis suggests that there are benefits to be gained from drawing on structures and knowledge that exist from population-related interventions and applying them to HIV interventions.

In conclusion, this analysis provides support for the hypothesis that organizational and structural features of countries, particularly those related to population interventions, facilitated better HIV outcomes. By focusing on macro factors, including population policy and family planning NGOs, this analysis highlights the importance of looking beyond the individual determinants of health outcomes to the structures that shape the context in which people live their lives, make decisions about health, and access health-related resources. It suggests that attention should be paid to building organizational and political structures that can assist in addressing HIV, but that can also be applied to future health needs. The analysis also indicates the importance of finding ways to address structural factors, such as poverty and cultural fractionalization, that inhibit positive outcomes.

## Competing interests

The author declares that she has no competing interests.

## Author’s contribution

All contributions are that of RSR as the sole author. She approved the final version of this manuscript.

## Author’s information

Rachel Sullivan Robinson is an assistant professor in the School of International Service at American University in Washington, DC Her research focuses on the politics of population, reproductive health and HIV/AIDS in sub-Saharan Africa, particularly as it relates to governments and non-governmental organizations. In addition, she has published on the determinants of fertility timing and outcomes in the journals, *Population Studies* and *Demography.* She is currently completing a book project, *Intimate Interventions*, that analyzes sex-related interventions in the realms of family planning and HIV in sub-Saharan Africa, and is centered on the cases of Senegal, Nigeria and Malawi.
